# A Rare Case of Digital Ischemia and Gangrene in ANCA-Associated Vasculitis with Review of the Literature

**DOI:** 10.1155/2017/2421760

**Published:** 2017-02-28

**Authors:** Richard A. Lau, Ramandeep Bains, Duminda Suraweera, Jane Ma, Emil R. Heinze, Andrew L. Wong, Philip J. Clements

**Affiliations:** ^1^UCLA-Olive View Rheumatology Program, Division of Rheumatology, Olive View-UCLA Medical Center, 14445 Olive View Drive, 2B182, Sylmar, CA 91342, USA; ^2^UCLA-Olive View Internal Medicine Program, Department of Medicine, Olive View-UCLA Medical Center, 14445 Olive View Drive, 2B182, Sylmar, CA 91342, USA

## Abstract

This paper describes one patient with Antineutrophil Cytoplasmic Antibody- (ANCA-) associated vasculitis who initially presented with multiple ischemic fingers and toes. On further evaluation, the patient was also found to have pulmonary-renal involvement and episcleritis. The diagnosis was supported with a positive cANCA (anti-proteinase 3) and a bronchoscopy consistent with diffuse alveolar hemorrhage. Although the patient refused a tissue biopsy, clinical presentation including nasal ulceration, sinus congestion, and epistaxis and anti-proteinase 3 antibody were more consistent with Granulomatosis with Polyangiitis (GPA) rather than Microscopic Polyangiitis (MPA) or Eosinophilic Granulomatosis with Polyangiitis (EGPA) based on the recently presented ACR/EULAR Provisional 2017 Classification Criteria for GPA (Luqmani et al., 2016). The patient responded well to therapy including high dose steroids and cyclophosphamide, with improvement of all organs involved and had no further digital ischemia or gangrene on follow-up. We include a review of the English literature summarizing presentation, management, and outcome of 16 similar cases.

## 1. Introduction

Antineutrophil Cytoplasmic Antibody- (ANCA-) associated vasculitis is a group of autoimmune small to medium vessel necrotizing vasculitides [1–6]. Granulomatosis with Polyangiitis (GPA), previously known as Wegener's, is a type of ANCA vasculitis that is usually associated with cANCA. The range of clinical manifestations of GPA can involve almost any organ system, but the classic organ systems involved include the upper respiratory tract, lower respiratory tract, and kidneys [1–6]. One of the rarer manifestations is digital ischemia and gangrene. We report a rare case of GPA presenting with digital ischemia and gangrene, with a review of the literature.

## 2. Case

A 62-year-old male with a past medical history of hypertension and vitiligo presented to the hospital with 2 months of worsening left 1st toe, left 3rd finger, and right 2nd, 3rd, and 4th finger pain, swelling, and discoloration. The patient was a mechanic and endorsed an inciting traumatic event from dropping a heavy tool to his left 3rd finger and left 1st toe but denied any preceding trauma to the digits on his right hand. Soon after the initiation of the pain and swelling, there was also associated discoloration, with blue/black color changes with gradual scab formation.

On further review of systems, the patient reported several weeks of diffuse joint pains, intermittent eye pain and red eyes, hemoptysis, epistaxis, nasal congestion, and weight loss (unable to quantify but has decreased several belt notches). The patient otherwise denied any Raynaud's phenomenon, skin tightening, oral ulcerations, genital ulceration, chest pain, shortness of breath, abdominal pain, hematuria, ear pain, sinus pain, fevers, chills, night sweats, or smoking history.

**Figure 1:**
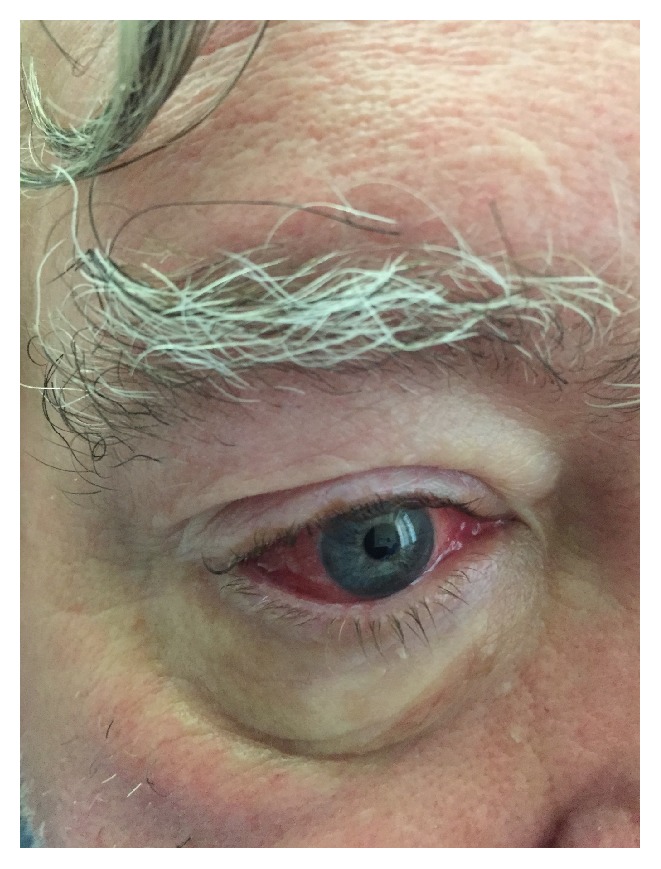


**Figure 2:**
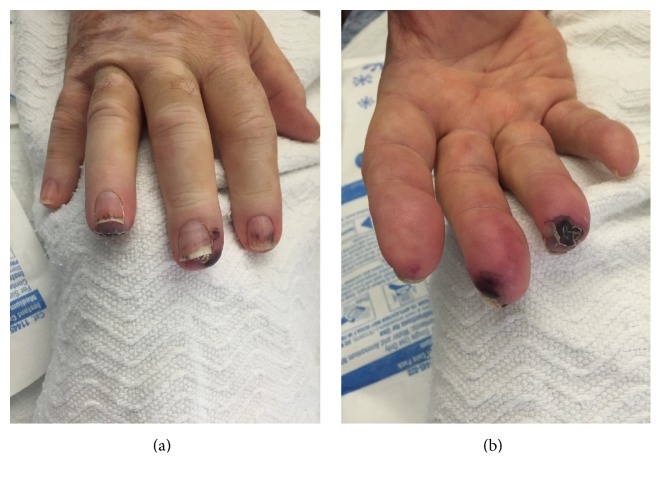


**Figure 3:**
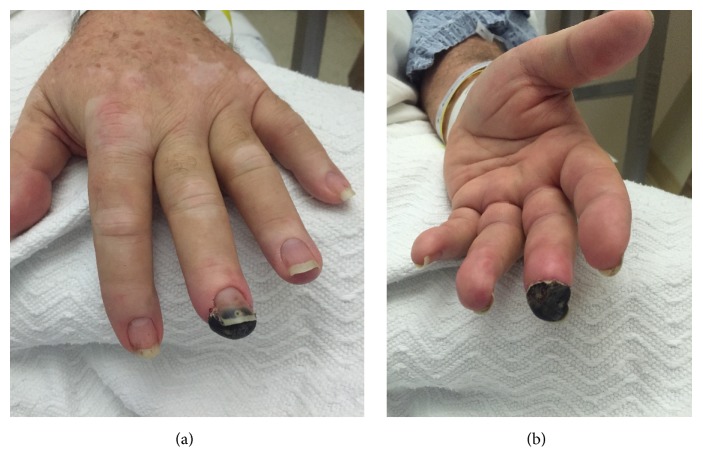


**Figure 4:**
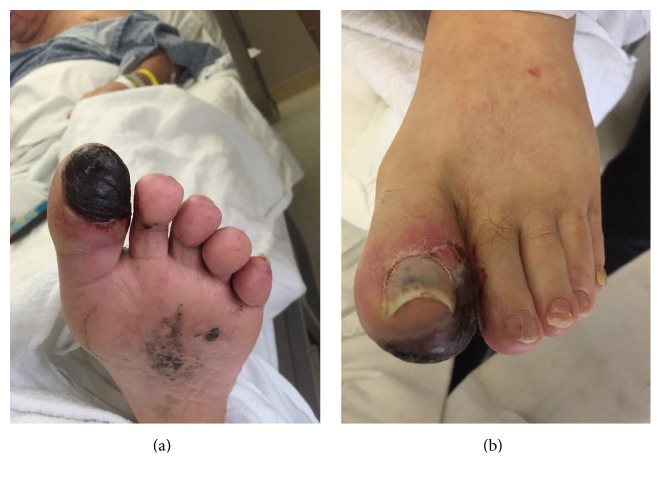


The patient's initial vitals were significant for hypertension only. On physical exam, the patient was in no acute distress. The head and neck exam was notable for bilateral conjunctivitis (see Figure 1) and right nasal ulceration. The cardiopulmonary exam was notable for poor airflow on lung auscultation. The skin exam was notable for vitiligo. The musculoskeletal exam was significant for diffuse joint tenderness but no joint swelling/warmth. The extremity exam revealed 3+ pitting edema of his lower extremities, right 2nd/3rd/4th finger duskiness and necrosis (see Figures 2(a) and 2(b)), left 3rd finger duskiness and necrosis (see Figures 3(a) and 3(b)), and left 1st toe with duskiness and necrosis (see Figures 4(a) and 4(b)).

The admitting labs were notable for elevated creatinine (1.74 mg/dL [ref 0.6–1.3]), elevated alkaline phosphatase (180 units/L [ref 38–126]), elevated CRP (140 mg/L [ref 0.0–7.0]), elevated ESR (67 mm/hr [ref < 20]), leukocytosis (12.5 thousand cells/uL [ref 3.8–10.9]), and anemia (12.5 gm/dL [ref 13.6–17.3]). Urinalysis revealed the presence of large blood, red blood cells (113 cells/HPF [ref 0–3]), white blood cells (13 cells/HPF [ref 0–5]), protein (100 mg/dL [ref negative]), and cellular casts (4/LPF). Random urine protein/creatinine ratio was 0.959 gm/gm. Initial chest X-ray on admission showed patchy right upper lobe airspace disease.

Given both pulmonary and renal system involvement, there was concern for pulmonary-renal syndromes, including ANCA-associated vasculitides, antiglomerular basement membrane disease, systemic lupus erythematous, and cryoglobulinemia. Other vasculitides of consideration included polyarteritis nodosum and thromboangiitis obliterans. We also considered that prothrombotic disorders such as antiphospholipid syndrome and embolic phenomenon can occur with cholesterol emboli or atrial myxoma. Infectious etiologies were considered as well, including endocarditis with embolic phenomenon. A paraneoplastic syndrome from a malignancy was entertained as well.

Further investigation was pursued to workup the aforementioned etiologies, including more laboratory evaluation with serologies pertinent for positive c-ANCA (proteinase-3 Ab 3.1 AI [ref < 1.0]), positive rheumatoid factor (80 IU/mL [ref < 14]), negative ANA, negative antiglomerular basement membrane antibody, negative anti-SSA/SSB, negative cryoglobulin screen, negative lupus anticoagulant, negative anti-Beta2 glycoprotein, and negative anticardiolipin. The infectious laboratory workup included multiple blood cultures with no growth. Transthoracic echocardiography did not show any thrombus, valvular disease, or atrial myxoma. Additional imaging was pursued that included computer tomography of the chest, which demonstrated extensive consolidation with air bronchograms predominately involving the right upper lobe and some contiguous involvement of the right middle and lower lobes. Bronchoscopy with serial bronchial washings by pulmonary consultants was consistent with alveolar hemorrhage. The ocular examination by ophthalmology consultants was consistent with episcleritis.

The patient was diagnosed with ANCA-associated vasculitis. The defining pathological difference between Granulomatosis with Polyangiitis (GPA) and Microscopic Polyangiitis (MPA) is the presence of granulomatous changes on biopsy based on our current classification criteria [1–3]. To support the diagnosis, we were planning on a skin biopsy but the patient refused. A renal biopsy was scheduled but the patient did not show for the procedure. Often even if biopsies are performed, there is a risk of missing definitive granuloma tissue due to sampling error, which makes differentiating the type of ANCA-associated vasculitis difficult. Based on the recently presented ACR/EULAR Provisional 2017 Classification Criteria for GPA, the patient scores 4 points on clinical criteria given his bloody nasal discharge, sinonasal congestion, and red or painful eyes and scores 5 points on serologic criteria with a positive cANCA-PR3 antibody. A combined score of greater than or equal to 5 is needed for classification of GPA [21]. The patient was classified as most likely having GPA and treated with IV methylprednisolone followed by oral prednisone, which was slowly tapered over the next several months. He was also started on monthly IV cyclophosphamide for a total of 6 months. The patient responded well to therapy with improvement of all of his organ dysfunction, including improvement of the reversible tissue ischemia, cessation of digital gangrenous extension, and eventual autoamputation of the irreversible gangrenous parts of his digits.

## 3. Discussion

Based on our review of the English literature, to the best of our knowledge, only 16 cases have been previously reported describing adult patients with GPA who presented with digital ischemia and gangrene [7–20] (see Table 1). Given the paucity of cases reported, we suspect the prevalence with digital ischemia and gangrene in the GPA population to be <1%. All of the cases in this review, including our case, satisfy the ACR/EULAR Provisional 2017 Classification Criteria for GPA with the exception of the 58-year-old male, patient number 17, in the case series by Pinching et al. where not enough specific case details were reported to confirm the diagnosis. The mean age of patients was 46.9 years and included a range of 24–80 years. Including our patient, 12 of 17 were males (71%) compared to 5 of 17 females (29%). Most patients presented with multiorgan involvement including 14 of 17 with pulmonary involvement (82%), 11 of 17 with kidney disease (65%), and 13 of 17 with sinonasal disease (76%). cANCA was confirmed positive in 13 patients and negative in one patient, and the data was not available in 3 of the cases. Only 3 patients demonstrated granulomas on biopsy. Confounding risk factors were inconsistently reported but include a past medical history of HTN in three cases [9, 16, 19], DM in three cases [13, 15, 16], tobacco use in two cases [13, 18], and dyslipidemia in one case [13]. The clinical phenotype usually involves multiple disparate digits. 10 of 17 cases described isolated involvement of the upper extremities (59%) compared to the 5 of 17 with isolated involvement of the lower extremities (29%). Only two cases (12%), including ours, have described simultaneous involvement of both the upper and lower extremities [20]. The pathophysiology of the digital ischemia and gangrene is thought to be from destruction of medium sized vessels from active vasculitis, which has been demonstrated in case reports where histological examination of affected tissue showed arteritis [9, 15]. However, there is also evidence that in situ thrombosis, as a result of active vasculitis, can lead to ischemia and gangrene, as seen in angiographic examination of affected patients, as well as on histological examination of affected tissue showing arterial thrombi [12, 13]. Initial symptoms in most cases included pain and swelling with eventual gangrene formation if left untreated. Digital ischemia and gangrene can be part of the initial presentation of GPA or develop later in the course of the disease, even while the patient is on active treatment. The diagnosis is usually made based on clinical grounds, but angiographic examination can support vasculitic or thrombotic lesions [7–20]. Pathology can demonstrate either vascular arteritis or thrombi formation [9, 12, 13, 15]. There is no consensus on treatment regarding GPA with digital vasculitis with ischemia and gangrene. However, cyclophosphamide and steroids are the most commonly used treatment in the literature [7–20]. Other therapies that have been used include anticoagulation and vasodilators. Surgical intervention with thrombectomy and bypass have been used as well.

Digital ischemia and gangrene are rare manifestations of GPA. They can have a heterogeneous presentation as demonstrated by the cases in the literature. Diagnosis can be difficult as biopsy of the skin is often nonspecific. Without treatment, ischemia and gangrene can progress and lead to significant disability. Therefore, it is important for clinicians to be aware of this rare manifestation and institute early treatment as indicated.

## Supplementary Material

Included are slides from the presentation at the 2016 ACR Annual Meeting outlining the new ACR-EULAR classification criteria for ANCA-associated vasculitis. Please note the ACR-EULAR Provisional 2017 Classification Criteria for Granulomatosis with Polyangiitis (GPA) on page 13.

## Figures and Tables

**Table 1:** Literature review of digital ischemia and gangrene associated with GPA in adults.

Age/sex	Onset after initial GPA symptoms	cANCA	Biopsy status	Other organ systems involved	Management	Outcome	Reference
30/M	3-4 months	Positive	Skin: nonspecific chronic inflammation Renal: segmental necrotizing GN	Sinusitis, epistaxis, arthralgias, painless oral ulcers, active urine sediment, mild renal insufficiency (creat. 2.3 mg/dL), and left upper lung lobe fibrotic infiltrates	Cyclophosphamide and steroids	Gangrene formation with autoamputation	Handa and Wali [7]

80/M	1-2 years	Positive	Skin: leukocytoclastic vasculitis	Multiple lung opacities with incomplete cavitation, renal insufficiency (creat. 1.5 mg/dL), microscopic hematuria, sensorineural hearing loss, and nasal ulcers	Cyclophosphamide and steroids	Gangrene formation, improvement of reversible tissue ischemia	La Civita et al. [8]

68/M	2 years	Not available	Sural nerve: vasculitis with secondary demyelination Lung: nodular necrotizing granulomatous inflammation with vasculitis	Purulent sinusitis, renal insufficiency (creat. 1.7 mg/dL), right peroneal neuropathy, otitis media, right 7th nerve palsy, pulmonary nodules, left pleural effusion, and pyoderma gangrenosum	Cyclophosphamide and steroids	Gangrene formation with surgical debridement and amputation	Phillips and Twiest [9]

26/F	1-2 years	Positive	Renal: focal crescentic GN Nasopharyngeal: small artery vasculitis	Secretory otitis, conjunctivitis, tonsillitis, arthritis, hematuria, mild renal insufficiency, mucosal ulcerations, and palpable purpura	Not specified	Gangrene formation	Karjalainen and Hakala [10]

48/M	3 months	Positive	Renal: pauci immune GN	Acute kidney injury (creat. 4.6 mg/dL), nasal crusting, hemoptysis, right sided pleuritic chest pain, and active urine sediment	Cyclophosphamide, steroids, heparin, aspirin, calcium channel blockers, nitroglycerin ointment, and plasmapheresis	Gangrene formation with autoamputation	Lim et al. [11]

24/F	On presentation	Positive	Femoral artery: normal	Pulmonary infiltrates, splenic infarcts, hemoptysis, and nodular masses in bilateral lungs with diffuse interstitial lung disease	Cyclophosphamide, steroids, heparin, and thrombectomy	Gangrene formation with surgical amputation	Bessias et al. [12]

55/M	3-4 months	Negative	Nasopharyngeal: epithelial ulceration with vasculitis	Epistaxis, polyarthralgia, bilateral lung nodular densities with cavitations, and small pleural effusion	Cyclophosphamide, steroids, warfarin, thrombectomy, and bypass	Improvement of reversible tissue ischemia	Maia et al. [13]

58/M	Unclear	Not available	Renal: 30% crescents	ENT, sinusitis, bronchiectasis, lung infiltrates, renal insufficiency (creat. 4.7 mg/dL), eye, and joint pain	Immunosuppression (not specified)	Gangrene formation	Pinching et al. [14]

40/M	Unclear	Not available	Renal: granuloma and vasculitis with 30% crescents Respiratory tract: vasculitis	ENT, lung infiltrates, renal failure requiring hemodialysis (creat. 18.43 mg/dL), joint pain, eye, vasculitic skin rash, and mononeuritis multiplex	Immunosuppression (not specified)	Gangrene formation	Pinching et al. [14]

39/F	On presentation	Positive	Lung: patchy nodular fibrotic changes with hemosiderin laden macrophages and diffuse alveolar hemorrhage Left 4th gangrene digit: vasculitic changes with no thrombotic phenomenon	Hemoptysis, decline in respiratory function, and bilateral pulmonary infiltrates	Cyclophosphamide, steroids, and plasmapheresis	Gangrene formation with surgical amputation, improvement of reversible tissue ischemia	Leung et al. [15]

45/M	2 years	Positive	Renal: necrotizing GN	Polyneuropathy, renal injury, arthralgia, mild bloody nasal discharge, and skin vasculitis	Cyclophosphamide, steroids, heparin, aspirin, and iloprost	Improvement of reversible tissue ischemia	Schmidt et al. [16]

41/M	2 years	Positive	Sinus: granulomatous vasculitis	Bloody nasal discharge, inner ear granuloma, polyneuropathy, pulmonary infiltrates, and destructive sinusitis	Cyclophosphamide, steroids, heparin, aspirin, and iloprost	Improvement of reversible tissue ischemia	Schmidt et al. [16]

49/M	On presentation	Positive	Sural nerve: axonal loss	Mononeuritis multiplex with left foot drop, and cavitary nodule in the lung	Cyclophosphamide, steroids	Acroosteolysis, improvement of reversible ischemia	Modi et al. [17]

61/F	4 months	Positive	Renal: focal segmental necrotizing GN	Joint pain, Raynaud's, and acute kidney injury	Cyclophosphamide, steroids	Gangrene formation with autoamputation	Bartsch et al. [18]

46/F	2 months	Positive	Skin: leukocytoclastic vasculitis	Polyarthralgias, epistaxis, sinusitis, mouth ulcers, episcleritis, mononeuritis multiplex, multiple nodules in right lung, and active urine sediment	Cyclophosphamide, steroids, and iloprost	Gangrene formation	Kejriwal et al. [19]

26/M	On presentation	Positive	Skin: superficial dermal blood vessel necrosis	Oligoarthritis, polydipsia, polyuria, lung nodules, and mucosal thickening of maxillary sinus	Cyclophosphamide, steroids, and azathioprine	Gangrene formation with autoamputation	Agarwal and Khan [20]

62/M	On presentation	Positive		Nasal ulceration and congestion, epistaxis, episcleritis, hemoptysis, diffuse alveolar hemorrhage, pulmonary infiltrates, joint pain, renal insufficiency (creat. 1.7), and active urine sediment	Cyclophosphamide, steroids	Improvement of reversible ischemia, gangrene formation with autoamputation	Current case

M: male; F: female; ENT: ear, nose, and throat; GN: glomerulonephritis; IV: intravenous.
