# Supplementary material for: A Rare Case of Digital Ischemia and Gangrene in ANCA-Associated Vasculitis with Review of the Literature
Source: Case Rep Rheumatol. 2017 Feb 28;2017:2421760. doi: 10.1155/2017/2421760 (PMC5350346; doi:10.1155/2017/2421760)
Supplement: Supplementary file 1 — Included are slides from the presentation at the 2016 ACR Annual Meeting outlining the new ACR-EULAR classification criteria for ANCA-associated vasculitis. Please note the ACR-EULAR Provisional 2017 Classification Criteria for Granulomatosis with Polyangiitis (GPA) on page 13. [file 2421760.f1.pdf]

DCVAS

New ACR-EULAR classification criteria for ANCA-associated vasculitis

Lead Investigators

Raashid Luqmani, University of Oxford
Peter A. Merkel, University of Pennsylvania
Richard Watts, University of East Anglia

Disclosures

- Ravi Suppiah – None
- Peter Grayson – None
- Cristina Ponte – None
- Jo Robson – None
- Richard Watts – None
- Peter Merkel – None
- Raashid Luqmani – None

Evidence based

Prior Classification/Nomenclature Schemes in Vasculitis

1. Leavitt RY et al. The American College of Rheumatology 1990 criteria for the classification of Wegener's granulomatosis. *Arthritis Rheum* 1990.
2. Masi AT et al. The American College of Rheumatology 1990 criteria for the classification of Churg-Strauss syndrome (allergic granulomatosis and angiitis). *Arthritis Rheum* 1990.
3. Lightfoot RW et al. The American College of Rheumatology 1990 criteria for the classification of polyarteritis nodosa. *Arthritis Rheum* 1990.
4. Jennette JC et al. Nomenclature of systemic vasculitides. Proposal of an international consensus conference. *Arthritis Rheum* 1994
5. Jennette JC et al. 2012 Revised International Chapel Hill Consensus Conference Nomenclature of Vasculitides. *Arthritis Rheum* 2013.

Acknowledgements

DCVAS is *sponsored* by the University of Oxford and supported in the UK by the NIHR Clinical Research Network

DCVAS is *funded* by the American College of Rheumatology, the European League Against Rheumatism and the Vasculitis Foundation

Introduction to the ACR-EULAR classification criteria for ANCA-associated vasculitis symposium

- Why and how?
  Ravi Suppiah, Peter Grayson, Cristina Ponte
- Results of 2017 Provisional classification criteria for Granulomatosis with Polyangiitis (GPA)
  Joanna Robson
- Implications for Clinical Practice / Q&A
  Raashid Luqmani, Peter Merkel, Richard Watts

DCVAS

New ACR-EULAR classification criteria for ANCA-associated vasculitis: *why and how?*

Presenters

Ravi Suppiah, Auckland District Health Board
Peter Grayson, United States National Institutes of Health
Cristina Ponte, Lisbon Academic Medical Centre

Overview of this section

1. **Why** do we need new criteria?

2. **How** did we develop new criteria?

1. Why?

Example case 1

- 45 year-old female
- Unwell for 2 weeks
- Fevers to 38°C (100°F)
- Weight loss of 4kg (8.8lb)
- Left foot and right wrist drop

1. Why?

Example case 1

- 45 year-old female
- Unwell for 2 weeks
- Fevers to 38°C (100°F)
- Weight loss of 4kg (8.8lb)
- Left foot and right wrist drop
- Scleritis

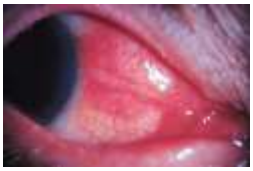

1. Why?

Example case 1

- 45 year-old female
- Unwell for 2 weeks
- Fevers to 38°C (100°F)
- Weight loss of 4kg (8.8lb)
- Left foot and right wrist drop
- Scleritis
- Progressive breathlessness for 4 months

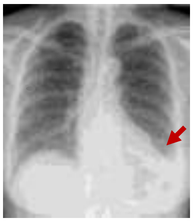

1. Why?

Example case 1

- 45 year-old female
- Unwell for 2 weeks
- Fevers to 38°C (100°F)
- Weight loss of 4kg (8.8lb)
- Left foot and right wrist drop
- Scleritis
- Progressive breathlessness for 4 months

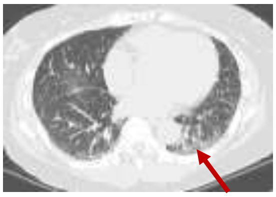

HRCT Chest

Fibrotic NSIP

1. Why?

Example case 1 - Investigations

- Hb 101 g/L (10.1g/dL)
- Eosinophil count normal
- CRP 180 mg/L
- Creatinine 360 umol/L (3.5 mg/dL)

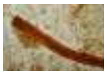

Red cell casts in urine

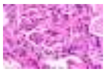

Pauci immune crescentic GN on renal biopsy

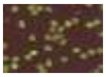

pANCA positive on IF

MPO-ANCA positive by ELISA

1. Why?

Example case 1 - How would you classify this case?

A. Polyarteritis nodosa (PAN)
B. Eosinophilic granulomatosis with polyangiitis (EGPA)
C. Granulomatosis with polyangiitis (GPA)
D. Microscopic polyangiitis (MPA)
E. Other type of vasculitis

1. Why?

Example case 1 - How would you classify this case?

A. Polyarteritis nodosa (PAN)
B. Eosinophilic granulomatosis with polyangiitis (EGPA)
C. Granulomatosis with polyangiitis (GPA)
D. Microscopic polyangiitis (MPA)
E. Other type of vasculitis

1. Why?

Example case 1 - How would you classify this case?

No microscopic polyangiitis (MPA) in the 1990 ACR classification criteria

1. Why?

Example case 1 - 1990 ACR Classification – GPA (Wegener's)

1. Nasal or oral inflammation
2. Abnormal chest radiograph
3. Urinary sediment
4. Granulomatous inflammation on biopsy

Requires 2 of the 4 criteria to be classified as GPA

Leavitt RY et al. *Arthritis Rheum* 1990

1. Why?

Example case 1 - 1990 ACR Classification – GPA (Wegener's)

1. Nasal or oral inflammation
2. **Abnormal chest radiograph**
3. **Urinary sediment**
4. Granulomatous inflammation on biopsy

Requires 2 of the 4 criteria to be classified as GPA

Patient meets criteria for GPA

Leavitt RY et al. *Arthritis Rheum* 1990

1. Why?

Example case 1 - 1990 ACR Classification - PAN

1. Weight loss ≥4 kg
2. Livedo reticularis
3. Testicular pain or tenderness
4. Myalgias, weakness, or leg tenderness
5. Mononeuropathy or polyneuropathy
6. Diastolic BP >90 mmHg
7. Elevated BUN or creatinine
8. Hepatitis B virus
9. Arteriographic abnormality
10. Biopsy of small or medium sized artery containing PMN

Requires 3 of the 10 criteria to be classified as PAN

Lightfoot RW et al. *Arthritis Rheum* 1990

1. Why?

Example case 1 - 1990 ACR Classification - PAN

1. **Weight loss ≥4 kg**
2. Livedo reticularis
3. Testicular pain or tenderness
4. Myalgias, weakness, or leg tenderness
5. **Mononeuropathy or polyneuropathy**
6. Diastolic BP >90 mmHg
7. **Elevated BUN or creatinine**
8. Hepatitis B virus
9. Arteriographic abnormality
10. Biopsy of small or medium sized artery containing PMN

Requires 3 of the 10 criteria to be classified as PAN

Patient meets criteria for PAN

Lightfoot RW et al. *Arthritis Rheum* 1990

1. Why?

Example case 2

- 73 year old female
- Unwell for 2 months
- Fevers to 38°C (100°F)
- Night sweats
- Weight loss of 3kg (6.6lb)
- Myalgia and arthralgia
- Cough

1. Why?

Example case 2

- 73 year old female
- Unwell for 2 months
- Fevers to 38°C (100°F)
- Night sweats
- Weight loss of 3kg (6.6lb)
- Myalgia and arthralgia
- Cough

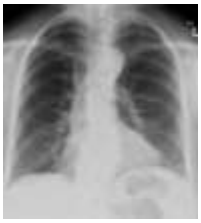

Normal CXR

1. Why?

Example case 2

- 73 year old female
- Unwell for 2 months
- Fevers to 38°C (100°F)
- Night sweats
- Weight loss of 3kg (6.6lb)
- Myalgia and arthralgia
- Cough

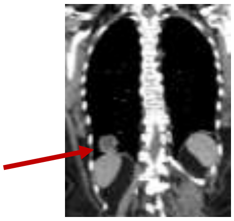

CT Chest–same day as CXR

1. Why?

Example case 2

- 73 year old female
- Unwell for 2 months
- Fevers to 38°C (100°F)
- Night sweats
- Weight loss of 3kg (6.6lb)
- Myalgia and arthralgia
- Cough

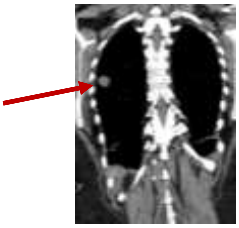

CT Chest–same day as CXR

1. Why?

Example case 2

- 73 year old female
- Unwell for 2 months
- Fevers to 38°C (100°F)
- Night sweats
- Weight loss of 3kg (6.6lb)
- Myalgia and arthralgia
- Cough

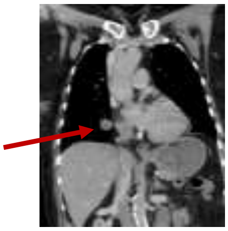

CT Chest–same day as CXR

1. Why?

Example case 2

- Sensation of blocked left ear with reduced hearing

- CRP 82 mg/L
- Urine normal
- Blood cultures negative
- Eosinophil count normal

1. Why?

Example case 2

- Sensation of blocked left ear with reduced hearing

- CRP 82 mg/L
- Urine normal
- Blood cultures negative
- Eosinophil count normal

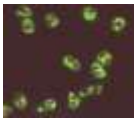

cANCA positive on IF

PR3-ANCA strongly positive on ELISA

1. Why?

Example case 2

- Sensation of blocked left ear with reduced hearing

- CRP 82 mg/L
- Urine normal
- Blood cultures negative
- Eosinophil count normal

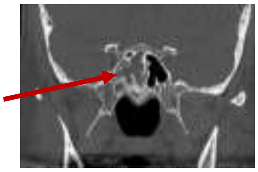

Opacified right sphenoid sinus

1. Why?

Example case 2 - How would you classify this case?

A. Polyarteritis nodosa (PAN)
B. Eosinophilic granulomatosis with polyangiitis (EGPA)
C. Granulomatosis with polyangiitis (GPA)
D. Microscopic polyangiitis (MPA)
E. Other type of vasculitis

1. Why?

Example case 2 - How would you classify this case?

A. Polyarteritis nodosa (PAN)
B. Eosinophilic granulomatosis with polyangiitis (EGPA)
C. Granulomatosis with polyangiitis (GPA)
D. Microscopic polyangiitis (MPA)
E. Other type of vasculitis

1. Why?

Example case 2 - 1990 ACR Classification – GPA (Wegener's)

1. Nasal or oral inflammation
2. Abnormal chest radiograph
3. Urinary sediment
4. Granulomatous inflammation on biopsy

Requires 2 of the 4 criteria to be classified as GPA

Leavitt RY et al. *Arthritis Rheum* 1990

1. Why?

Example case 2 - 1990 ACR Classification – GPA (Wegener's)

1. Nasal or oral inflammation
2. Abnormal chest radiograph
3. Urinary sediment
4. Granulomatous inflammation on biopsy

Requires 2 of the 4 criteria to be classified as GPA

Patient does **NOT** meet criteria for GPA

Leavitt RY et al. *Arthritis Rheum* 1990

1. Why?

Why do we need new criteria?

- Old criteria not consistent with current disease definitions
- No MPA
- ANCA
- Improved imaging

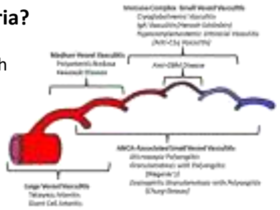

2012 Chapel Hill Consensus Conference

Jennette JC et al. *Arthritis Rheum* 2013

1. Why?

Why do we need new criteria?

- Old criteria not consistent with current disease definitions
- No MPA
- ANCA
- Improved imaging

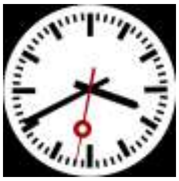

TIME FOR AN UPDATE

2. How?

Background to the methodology

Classification *vs* Diagnostic Criteria

- Classification criteria enable stratification of homogenous groups to standardize recruitment into clinical trials and related studies
- Distinguish amongst different types of vasculitis
  - No inclusion of vasculitis mimics as comparators

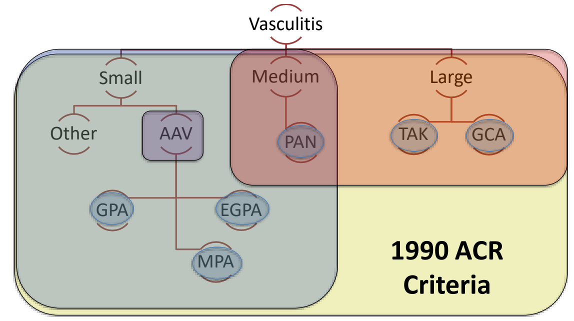

133 sites in 33 countries

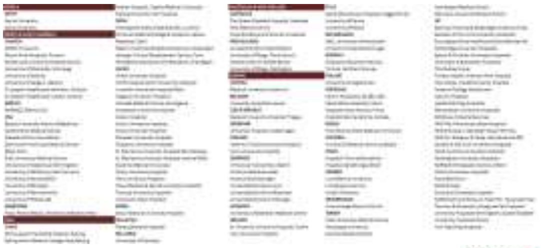

2. How?

Methodology Advancements

Number of Criteria Rule

Weighted Criterion with Threshold Score

DCVAS

2. How?

1987 ACR Rheumatoid Arthritis Classification Criteria

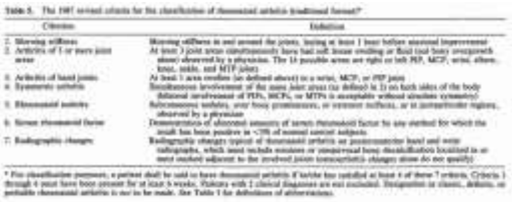

DCVAS

2. How?

2010 ACR/EULAR Rheumatoid Arthritis Classification Criteria

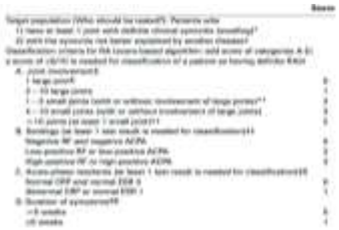

DCVAS

2. How?

Establishing the Gold Standard

Submitting Physician Diagnosis

Expert Panel Review

DCVAS

2. How?

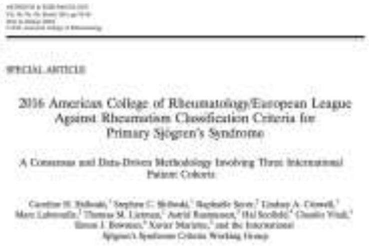

DCVAS

2. How?

DCVAS Classification criteria methodology: 3 phases

1. Case selection	• Clinical vignette expert panel review<br>• Avoid circularity of applying “gold standard”<br>• Quality control
2. Item selection	• Data-driven item reduction<br>• Clinical consensus item reduction
3. Final Criteria	• Combined data-driven and clinical expert review<br>• Model performance: face validity, discrimination

DCVAS

2. How?

1. Case selection

Production of clinical vignettes

2. How?

1. Case selection

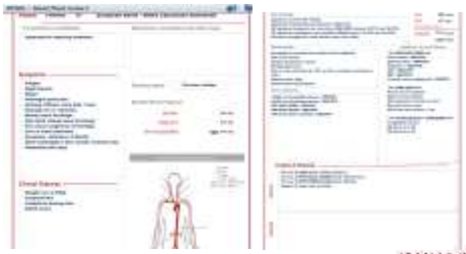

2. How?

1. Case selection

Production of clinical vignettes

Expert reviewers chosen from diverse geographical locations and specialties

↓

Web-based review system

2. How?

1. Case selection

Independent expert review of each clinical vignette

Expert reviewers were asked:

Vasculitis? → Vessel size? → ANCA-associated vasculitis? → Subtype?

- Level of certainty: **very certain**, **moderately certain**, **uncertain**, or **very uncertain**
- **Match on subtype** (≥ moderate certainty) - Case passed
- **No match** - Second review by DCVAS committee member

2. How?

2. Item selection

Item selection: data-driven and expert consensus

1000+ DCVAS data items (Clinical, Laboratory, Imaging and Biopsy)

↓

Assessed the items for GPA, MPA, EGPA and "other small vessel vasculitides"

Excluded items	Identified prevalence of individual items	
	< 5% prevalence	> 5% prevalence
• Normal/negative<br>• Low clinical relevance	Reviewed for clinical relevance and discrimination	

Reviewed by steering committee - retained as individual or combined items

Approximately 100 items for regression analysis

2. How?

3. Final criteria

Statistical methods

- Development (80%) and validation (20%) datasets
- Too many predictors for standard regression analysis, even after data reduction
- Specific type of analysis - Lasso logistic regression
- Multiple imputation for missing data

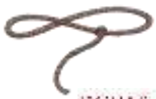

Menelaos Pavlou et al. *BMJ Research Methods and Reporting* 2015

Questions?

DCVAS

2017 Provisional Classification Criteria for Granulomatosis with Polyangiitis (GPA)

Presenter
Joanna Robson MBBS PhD MRCP
Consultant Senior Lecturer in Rheumatology
University of the West of England, Bristol

Results

DCVAS Classification criteria: 3 phases

1. Case selection
- Clinical vignette expert panel review
- Avoid circularity of applying “gold standard”
- Quality control

Expert reviewer characteristics

1. Case selection

- Forty-nine reviewers from 22 countries

Specialty

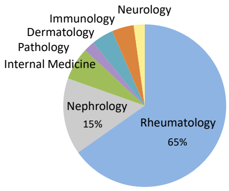

No. of AAV patients seen per year

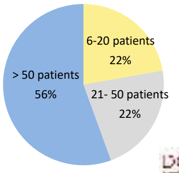

Expert reviewer process

1. Case selection

DCVAS cases with submitting physician diagnosis with high or moderate confidence (N=1496)

↓

49 external expert reviewers (~28 cases each)

Expert reviewer process

1. Case selection

DCVAS cases with submitting physician diagnosis with high or moderate confidence (N=1496)

↓

49 external expert reviewers (~28 cases each)

- Expert agree N=782 (53%)
- Expert no agreement N= 714 (48%)
  ↓
  7 DCVAS committee reviewers (~100 each)

1. Case selection

Expert reviewer process

DCVAS cases with submitting physician diagnosis with high or moderate confidence (N=1496)

49 external expert reviewers (~28 cases each)

Expert agree
N=782 (53%)

Expert no agreement
N= 714 (48%)

7 DCVAS committee reviewers (~100 each)

Agreement
PASSED N= 1111
(75% of all cases)

Committee agree
OR expert & DCVAS reviewer agree
N=329

No agreement
REJECTED N= 385
(25% of all cases)

1. Case selection

Expert review of cases

Type of vasculitis	Submitting physician cases	Total cases passed on expert review
Granulomatosis with polyangiitis	578	492
Microscopic polyangiitis	275	205
Eosinophilic granulomatosis with polyangiitis	214	156
AAV-cannot subtype	31	20
Anti-GBM	9	10
Cryoglobulinaemic vasculitis	32	24
IgA vasculitis	170	108
Other small vessel - cannot subtype	46	1
Polyarteritis nodosa	22	7
Giant Cell Arteritis	43	38
Takayasu arteritis	39	30
Vasculitis mimics	37	20

1. Case selection

Expert review of cases

Comparators for GPA

Type of vasculitis	Submitting physician cases	Total cases passed on expert review
**Granulomatosis with polyangiitis**	578	492
**Microscopic polyangiitis**	275	205
**Eosinophilic granulomatosis with polyangiitis**	214	156
**AAV-cannot subtype**	31	20
**Anti-GBM**	9	10
**Cryoglobulinaemic vasculitis**	32	24
**IgA vasculitis**	170	108
**Other small vessel - cannot subtype**	46	1
**Polyarteritis nodosa**	22	7
Giant Cell Arteritis	43	38
Takayasu arteritis	39	30
Vasculitis mimics	37	20

Results

DCVAS Classification criteria: 3 phases

1. Case selection
- Clinical vignette expert panel review
- Avoid circularity of applying “gold standard”
- Quality control

2. Item selection
- Data-driven item reduction
- Clinical consensus item reduction

2. Item selection

Item selection: data-driven and expert consensus

1000 DCVAS data items
(333 clinical and 276 laboratory items, 7830 imaging and 188 biopsy combinations)

Assessed the items for GPA, MPA, EGPA and “other small vessel vasculitides”

Excluded items and combinations: **8219**

Identified prevalence of items and combinations
- < 5% prevalence: **292**
- > 5% prevalence: **116**

Reviewed by steering committee

91 items retained for regression analysis
(45 clinical, 18 laboratory, 12 imaging and 16 biopsy combinations)

Results

DCVAS Classification criteria: 3 phases

1. Case selection
- Clinical vignette expert panel review
- Avoid circularity of applying “gold standard”
- Quality control

2. Item selection
- Data-driven item reduction
- Clinical consensus item reduction

3. Final Criteria
- Combined data-driven and clinical expert review
- Model performance: face validity, discrimination

3. Final criteria

Patient characteristics for GPA analysis

	GPA n = 492	Comparators n = 531	p-value
Age (SD)	53.4 (16.4)	57.8 (16.9)	<0.001
Gender (F)	(47.2% of 492)	(49.5% of 531)	0.32
Creatinine umol/L	159.3	203.0	<0.001
mg/dL	1.8	2.3	
c-ANCA positive (%)	72.4%	5.5%	<0.01
p-ANCA positive (%)	10.4%	47.3%	<0.01
PR3- antibody positive (%)	80.9%	3.4%	<0.01
MPO- antibody positive (%)	8.30%	53.9%	<0.01

DCVAS

3. Final criteria

Data split of development and validation sets (80% : 20%)

	Development set	Validation set
GPA	395 (48.2%)	97 (47.5%)
Comparators	424 (51.8%)	107 (52.5%)
Total	819	204

DCVAS

3. Final criteria

Results (all 22 independent variables)

Rank	Description	OR (95% CI)
1	cANCA or PR3	59.3 (22.2, 158.4)
2	Bloody nasal discharge	18.1 (6.00, 54.6)
3	Nasal ulcers, crusting or sino nasal congestion or blockage	5.79 (2.28, 14.7)
4	Eosinophil count ≥ 1 x10⁹/L	0.05 (0.02, 0.17)
5	Nasal polyps	0.03 (0.00, 0.23)
6	Nodules/ mass or cavitation on chest imaging	7.44 (2.76, 20.0)
7	Hearing loss or reduction	5.90 (1.96, 17.8)
8	IgA deposition on biopsy	0.02 (0.00, 0.25)
9	Granuloma on biopsy	5.48 (1.30, 23.1)
10	pANCA or MPO	0.25 (0.10, 0.64)
11	Red or painful eyes	3.87 (1.15, 13.0)

Rank	Description	OR (95% CI)
12	Cartilagenous involvement	3.96 (1.03, 15.2)
13	Abdominal pain	0.40 (0.13, 1.25)
14	Obstructive airways	0.18 (0.06, 0.59)
15	Mononeuritis multiplex OR Motor neuropathy (not radiculopathy)	0.30 (0.12, 0.78)
16	Anaemia (Hb <10g/dL)	0.36 (0.16, 0.79)
17	Non-productive cough	2.89 (1.12, 7.50)
18	Inflammation, consolidation or effusion of the nasal/ paranasal sinuses on imaging	1.39 (0.47, 4.09)
19	Non blood stained nasal discharge	2.01 (0.61, 6.67)
20	Fever, night sweats or rigors	2.54 (1.16, 5.55)
21	Fibrosis or ILD on imaging of the chest	0.11 (0.02, 0.68)
22	Fatigue	0.54 (0.25, 1.17)

DCVAS

3. Final criteria

Results (all 22 independent variables)

Data driven only top 5

Rank	Description	OR (95% CI)
1	cANCA or PR3	59.3 (22.2, 158.4)
2	Bloody nasal discharge	18.1 (6.00, 54.6)
3	Nasal ulcers, crusting or sino nasal congestion or blockage	5.79 (2.28, 14.7)
4	Eosinophil count ≥ 1 x10⁹/L	0.05 (0.02, 0.17)
5	Nasal polyps	0.03 (0.00, 0.23)
6	Nodules/ mass or cavitation on chest imaging	7.44 (2.76, 20.0)
7	Hearing loss or reduction	5.90 (1.96, 17.8)
8	IgA deposition on biopsy	0.02 (0.00, 0.25)
9	Granuloma on biopsy	5.48 (1.30, 23.1)
10	pANCA or MPO	0.25 (0.10, 0.64)
11	Red or painful eyes	3.87 (1.15, 13.0)

Rank	Description	OR (95% CI)
12	Cartilagenous involvement	3.96 (1.03, 15.2)
13	Abdominal pain	0.40 (0.13, 1.25)
14	Obstructive airways	0.18 (0.06, 0.59)
15	Mononeuritis multiplex OR Motor neuropathy (not radiculopathy)	0.30 (0.12, 0.78)
16	Anaemia (Hb <10g/dL)	0.36 (0.16, 0.79)
17	Non-productive cough	2.89 (1.12, 7.50)
18	Inflammation, consolidation or effusion of the nasal/ paranasal sinuses on imaging	1.39 (0.47, 4.09)
19	Non blood stained nasal discharge	2.01 (0.61, 6.67)
20	Fever, night sweats or rigors	2.54 (1.16, 5.55)
21	Fibrosis or ILD on imaging of the chest	0.11 (0.02, 0.68)
22	Fatigue	0.54 (0.25, 1.17)

DCVAS

3. Final criteria

Results (all 22 independent variables)

Data driven only top 5

Rank	Description	OR (95% CI)
1	cANCA or PR3	59.3 (22.2, 158.4)
2	Bloody nasal discharge	18.1 (6.00, 54.6)
3	Nasal ulcers, crusting or sino nasal congestion or blockage	5.79 (2.28, 14.7)
4	Eosinophil count ≥ 1 x10⁹/L	0.05 (0.02, 0.17)
5	Nasal polyps	0.03 (0.00, 0.23)
6	Nodules/ mass or cavitation on chest imaging	7.44 (2.76, 20.0)
7	Hearing loss or reduction	5.90 (1.96, 17.8)
8	IgA deposition on biopsy	0.02 (0.00, 0.25)
9	Granuloma on biopsy	5.48 (1.30, 23.1)
10	pANCA or MPO	0.25 (0.10, 0.64)
11	Red or painful eyes	3.87 (1.15, 13.0)

Rank	Description	OR (95% CI)
12	Cartilagenous involvement	3.96 (1.03, 15.2)
13	Abdominal pain	0.40 (0.13, 1.25)
14	Obstructive airways	0.18 (0.06, 0.59)
15	Mononeuritis multiplex OR Motor neuropathy (not radiculopathy)	0.30 (0.12, 0.78)
16	Anaemia (Hb <10g/dL)	0.36 (0.16, 0.79)
17	Non-productive cough	2.89 (1.12, 7.50)
18	Inflammation, consolidation or effusion of the nasal/ paranasal sinuses on imaging	1.39 (0.47, 4.09)
19	Non blood stained nasal discharge	2.01 (0.61, 6.67)
20	Fever, night sweats or rigors	2.54 (1.16, 5.55)
21	Fibrosis or ILD on imaging of the chest	0.11 (0.02, 0.68)
22	Fatigue	0.54 (0.25, 1.17)

DCVAS

3. Final criteria

Results (all 22 independent variables)

Rank	Description	OR (95% CI)
1	cANCA or PR3	59.3 (22.2, 158.4)
2	Bloody nasal discharge	18.1 (6.00, 54.6)
3	Nasal ulcers, crusting or sino nasal congestion or blockage	5.79 (2.28, 14.7)
4	Eosinophil count ≥ 1 x10⁹/L	0.05 (0.02, 0.17)
5	Nasal polyps	0.03 (0.00, 0.23)
6	Nodules/ mass or cavitation on chest imaging	7.44 (2.76, 20.0)
7	Hearing loss or reduction	5.90 (1.96, 17.8)
8	IgA deposition on biopsy	0.02 (0.00, 0.25)
9	Granuloma on biopsy	5.48 (1.30, 23.1)
10	pANCA or MPO	0.25 (0.10, 0.64)
11	Red or painful eyes	3.87 (1.15, 13.0)

Rank	Description	OR (95% CI)
12	Cartilagenous involvement	3.96 (1.03, 15.2)
13	Abdominal pain	0.40 (0.13, 1.25)
14	Obstructive airways	0.18 (0.06, 0.59)
15	Mononeuritis multiplex OR Motor neuropathy (not radiculopathy)	0.30 (0.12, 0.78)
16	Anaemia (Hb <10g/dL)	0.36 (0.16, 0.79)
17	Non-productive cough	2.89 (1.12, 7.50)
18	Inflammation, consolidation or effusion of the nasal/ paranasal sinuses on imaging	1.39 (0.47, 4.09)
19	Non blood stained nasal discharge	2.01 (0.61, 6.67)
20	Fever, night sweats or rigors	2.54 (1.16, 5.55)
21	Fibrosis or ILD on imaging of the chest	0.11 (0.02, 0.68)
22	Fatigue	0.54 (0.25, 1.17)

DCVAS

3. Final criteria

Results (all 22 independent variables)

Clinical and data driven

Rank	Description	OR (95% CI)
1	cANCA or PR3	59.3 (22.2, 158.4)
2	Bloody nasal discharge	18.1 (6.00, 54.6)
3	Nasal ulcers, crusting or sino nasal congestion or blockage	5.79 (2.28, 14.7)
4	Eosinophil count ≥ 1 x10⁹/L	0.05 (0.02, 0.17)
5	Nasal polyps	0.03 (0.00, 0.23)
6	Nodules/ mass or cavitation on chest imaging	7.44 (2.76, 20.0)
7	Hearing loss or reduction	5.90 (1.96, 17.8)
8	IgA deposition on biopsy	0.02 (0.00, 0.25)
9	Granuloma on biopsy	5.48 (1.30, 23.1)
10	pANCA or MPO	0.25 (0.10, 0.64)
11	Red or painful eyes	3.87 (1.15, 13.0)

Clinical and data driven

Rank	Description	OR (95% CI)
12	Cartilagenous involvement	3.96 (1.03, 15.2)
13	Abdominal pain	0.40 (0.13, 1.25)
14	Obstructive airways	0.18 (0.06, 0.59)
15	Mononeuritis multiplex OR Motor neuropathy (not radiculopathy)	0.30 (0.12, 0.78)
16	Anaemia (Hb <10g/dL)	0.36 (0.16, 0.79)
17	Non-productive cough	2.89 (1.12, 7.50)
18	Inflammation, consolidation or effusion of the nasal/ paranasal sinuses on imaging	1.39 (0.47, 4.09)
19	Non blood stained nasal discharge	2.01 (0.61, 6.67)
20	Fever, night sweats or rigors	2.54 (1.16, 5.55)
21	Fibrosis or ILD on imaging of the chest	0.11 (0.02, 0.68)
22	Fatigue	0.54 (0.25, 1.17)

DCVAS

3. Final criteria

Data-driven and clinically selected 9 item model

Description	Odds Ratio (95%CI)	P-value
cANCA or PR3	134.8 (62.4, 291.1)	<0.001
Bloody nasal discharge/ ulcers/ crusting or sino-nasal congestion	16.0 (7.90, 32.2)	<0.001
Granuloma on biopsy	12.9 (3.76, 44.4)	<0.001
Nodules/ mass or cavitation on chest imaging	5.77 (2.63, 12.7)	<0.001
Cartilagenous involvement*	5.50 (1.87, 16.2)	<0.01
Red or painful eyes	4.42 (1.67, 11.7)	<0.01
Hearing loss or reduction	3.74 (1.60, 8.73)	<0.01
Maximum eosinophil count (x10⁹/L) (≥1 vs. <1)	0.08 (0.03, 0.19)	<0.001
Nasal polyps	0.02 (0.01, 0.10)	<0.001

*Inflamed ear or nose cartilage or hoarse voice/stridor, endobronchial involvement or saddle nose deformity

DCVAS

3. Final criteria

Points based risk score

Risk factor at baseline	Coefficient	Risk score
cANCA or PR3	4.90	+ 5
Bloody nasal discharge/ ulcers or crusting or sino-nasal congestion	2.77	+ 3
Granuloma on biopsy	2.56	+ 3
Nodules/ mass or cavitation on chest imaging	1.75	+ 2
Cartilagenous involvement	1.71	+ 2
Red or painful eyes	1.49	+ 1
Hearing loss or reduction	1.32	+ 1
Maximum eosinophil count ≥ 1 (x10⁹/L)	- 2.56	- 3
Nasal polyps	- 3.70	- 4

DCVAS

3. Final criteria

Model Performance

Model Performance	Development	Validation
**AUC (95% CI)**	0.98 (0.97, 0.99)	0.98 (0.96, 0.99)
**Cut-off ≥ 5 points**		
Sensitivity	89.9%	90.7%
Specificity	94.1%	93.5%
**Cut-off ≥ 6 points**		
Sensitivity	78.2%	75.3%
Specificity	98.4%	99.1%

DCVAS

3. Final criteria

Discrimination curves

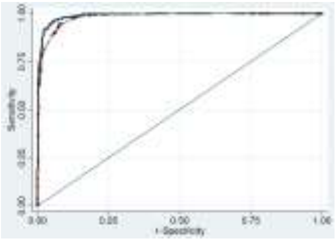

22 variables model (AUC 0.99)

9 variables model (AUC 0.98)

Risk Score (AUC 0.98)

DCVAS

ACR/ EULAR Provisional 2017 Classification Criteria for Granulomatosis with Polyangiitis (GPA)

This criteria set is for use when a diagnosis of small or medium vessel vasculitis has been made, in order to classify the patient as having GPA

	Item	Score
Clinical	Bloody nasal discharge, ulcers, crusting or sino-nasal congestion	3
	Nasal polyps	− 4
	Hearing loss or reduction	1
	Cartilagenous involvement	2
	Red or painful eyes	1
Tests	cANCA or PR3- antibody positive	5
	Eosinophil count ≥ 1 (x10⁹/L)	− 3
	Nodules, mass or cavitation on chest imaging	2
	Granuloma on biopsy	3

Add scores for 9 items; a score of ≥ 5 is needed for classification of GPA

DCVAS

Acknowledgements

Expert panel reviewers

Alojzija Hocevar
Aman Sharma
Andrea Hinojosa
Augusto Vaglio
Bernhard Hellmich
Bo Baslund
Carla Macieira
Carlo Salvarani
Carol Langford
Cees Kallenberg
Charles Jennette
Cord Sunderkötter
David Jayne
Eamonn Molloy
Eric Matteson
Haner Direskeneli
Jan Sznajd
Jason Springer
Joerg Henes
Julia Holle
Kazuo Matsui
Lillian Barra
Liza Rajasekhar
Loic Guillevin
Luis Felipe Flores-Suárez
Marco A Alba
Maria Cid
Mark Little
Nader Khalidi
Nataliya Milman
Neil Basu
Nevin Hammam
Nina Brown
Ömer Karadağ
Ora Gewurz-Singer
Paul A Monach
Paul Gatenby
Pavel Novikov
Robert Micheletti
Rona Smith
Ruth Geraldes
Sergey Moiseev
Shouichi Fujimoto
Thomas Daikeler
Thomas Hauser
Thomas Neumann
Vicky Quincey
Vladimir Tesar
Wolfgang Schmidt

Acknowledgements

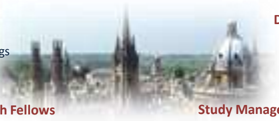

Statisticians
Andrew Judge
Andrew Hutchings
Sara Khalid

Data management
Ann-Marie Morgan
David Gray
Issi Platt

Clinical Research Fellows
Alberto Flores
Ana Rodrigues
Ben Seeliger
Hannah Querin
Jan Sznajd
Kasia Wawrzycka-Adamczyk
Sara Monti
Takahiro Nunokawa

Study Management
Anthea Craven

Site communications & payments
Marian Montgomery

Database design
Joe Barrett, Joe Rosa, Nathanael Gray

Questions?

DCVAS

New ACR/EULAR Classification Criteria for ANCA-Associated Vasculitis: *Implications for Clinical Practice*

Presenters

Raashid Luqmani, University of Oxford
Peter A. Merkel, University of Pennsylvania
Richard Watts, University of East Anglia

ACR/ EULAR Provisional 2017 Classification Criteria for Granulomatosis with Polyangiitis (GPA)

This criteria set is for use when a diagnosis of small or medium vessel vasculitis has been made, in order to classify the patient as having GPA

Clinical	Bloody nasal discharge, ulcers, crusting or sino-nasal congestion	3
Clinical	Nasal polyps	−4
Clinical	Hearing loss or reduction	1
Clinical	Cartilagenous involvement	2
Clinical	Red or painful eyes	1
Tests	cANCA or PR3- antibody positive	5
Tests	Eosinophil count ≥ 1 ($x10^9$/L)	−3
Tests	Nodules, mass or cavitation on chest imaging	2
Tests	Granuloma on biopsy	3

Add scores for 9 items; a score of ≥ 5 is needed for classification of GPA

Key points

Provide modern criteria for 21st century

- Clinically based
- Reflects current investigative practice
- Includes, BUT not critically dependent on
  - ANCA
  - Biopsy
- Provide a risk score

- Good specificity and sensitivity

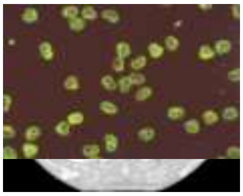

Clinical Implications

- Clinical trials based on modern classification criteria for ANCA-associated vasculitis
- Improvement in evidence base for decisions about therapy

Example Case 1

- 45 year-old woman
- Constitutional symptoms
- Left foot and right wrist drop
- Interstitial lung disease

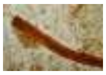
Red cell casts in urine

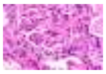
Pauci immune crescentic GN on renal biopsy

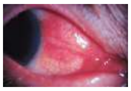
Scleritis

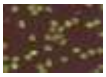
pANCA positive on IF

MPO-ANCA positive by ELISA

Example Case 1

Bloody nasal discharge, ulcers or crusting or sino-nasal congestion	3
Nasal polyps	− 4
Hearing loss or reduction	1
Cartilagenous involvement	2
**Red or painful eyes**	**1**
cANCA or PR3- antibody positive	5
Eosinophil count ≥ 1 (x10^9/L)	− 3
Nodules, mass or cavitation on chest imaging	2
Granuloma on biopsy	3

Risk score = 1; classified as *not* GPA

(score of ≥ 5 is needed for classification of GPA)

Example Case 2

- 73 year-old woman
- Constitutional symptoms
- Cough
- Reduced hearing

- CRP 82 mg/L
- Eosinophil count normal

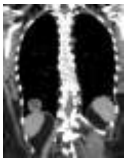
Normal CXR, but CT chest showing cavitating nodules

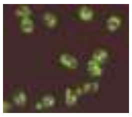
cANCA positive on IF

PR3-ANCA strongly positive on ELISA

Example Case 2

Bloody nasal discharge, ulcers or crusting or sino-nasal congestion	3
Nasal polyps	− 4
**Hearing loss or reduction**	**1**
Cartilagenous involvement	2
Red or painful eyes	1
**cANCA or PR3- antibody positive**	**5**
Eosinophil count ≥ 1 (x10^9/L)	− 3
**Nodules, mass or cavitation on chest imaging**	**2**
Granuloma on biopsy	3

Risk score = 8; classified as GPA

(score of ≥ 5 is needed for classification of GPA)

ACR-EULAR 2017 GPA criteria

Impact on the two cases

Case 1

- ACR 1990: Classified as PAN *and* GPA (WG)
- ACR/EULAR 2017: Risk score = 1; Not classified as GPA

Case 2

- ACR 1990: Not classified as GPA (WG)
- ACR/EULAR 2017: Risk score = 8; Classified as GPA

Future Analyses

- Develop criteria for AAV, PAN, TAK, GCA, and IgA
- Performance in different datasets
  e.g.
  – physician-submitted cases
  – external datasets
- Comparison with previous ACR criteria
- Overall clinical features of each group and outcomes
- Diagnostic criteria

DCVAS: Recruitment by Diagnosis

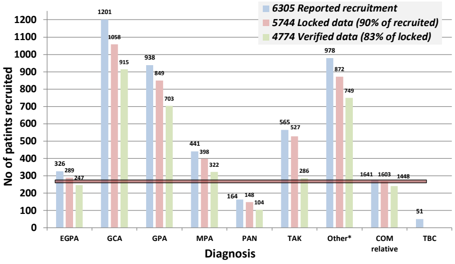

Questions?
